# Biochemical Assessment of Precuneus and Posterior Cingulate Gyrus in the Context of Brain Aging and Alzheimer’s Disease

**DOI:** 10.1371/journal.pone.0105784

**Published:** 2014-08-28

**Authors:** Chera L. Maarouf, Tyler A. Kokjohn, Douglas G. Walker, Charisse M. Whiteside, Walter M. Kalback, Alexis Whetzel, Lucia I. Sue, Geidy Serrano, Sandra A. Jacobson, Marwan N. Sabbagh, Eric M. Reiman, Thomas G. Beach, Alex E. Roher

**Affiliations:** 1 The Longtine Center for Neurodegenerative Biochemistry, Banner Sun Health Research Institute, Sun City, Arizona, United States of America; 2 Department of Microbiology, Midwestern University, Glendale, Arizona, United States of America; 3 Laboratory of Neuroinflammation, Banner Sun Health Research Institute, Sun City, Arizona, United States of America; 4 Civin Laboratory for Neuropathology, Banner Sun Health Research Institute, Sun City, Arizona, United States of America; 5 Cleo Roberts Center for Clinical Research, Banner Sun Health Research Institute, Sun City, Arizona, United States of America; 6 Banner Alzheimer’s Institute, Phoenix, Arizona, United States of America; Univ. Kentucky, United States of America

## Abstract

Defining the biochemical alterations that occur in the brain during “normal” aging is an important part of understanding the pathophysiology of neurodegenerative diseases and of distinguishing pathological conditions from aging-associated changes. Three groups were selected based on age and on having no evidence of neurological or significant neurodegenerative disease: 1) young adult individuals, average age 26 years (n = 9); 2) middle-aged subjects, average age 59 years (n = 5); 3) oldest-old individuals, average age 93 years (n = 6). Using ELISA and Western blotting methods, we quantified and compared the levels of several key molecules associated with neurodegenerative disease in the precuneus and posterior cingulate gyrus, two brain regions known to exhibit early imaging alterations during the course of Alzheimer’s disease. Our experiments revealed that the bioindicators of emerging brain pathology remained steady or decreased with advancing age. One exception was S100B, which significantly increased with age. Along the process of aging, neurofibrillary tangle deposition increased, even in the absence of amyloid deposition, suggesting the presence of amyloid plaques is not obligatory for their development and that limited tangle density is a part of normal aging. Our study complements a previous assessment of neuropathology in oldest-old subjects, and within the limitations of the small number of individuals involved in the present investigation, it adds valuable information to the molecular and structural heterogeneity observed along the course of aging and dementia. This work underscores the need to examine through direct observation how the processes of amyloid deposition unfold or change prior to the earliest phases of dementia emergence.

## Introduction

Aging comprises a series of inevitable time-dependent and progressive molecular changes. The rate at which an individual ages is genetically determined, but is also powerfully modulated by the cumulative effects of disease as well as complex environmental and behavioral factors. Aging is the most important risk factor for the development of neurodegenerative diseases and in particular for sporadic Alzheimer’s disease (AD), the most common form of dementia. An unintended consequence of the impressive enhancement in average life expectancy has been a dramatic increase in AD prevalence.

The precuneus (Pc) and posterior cingulate gyrus (PCG) of the cerebral cortex are regions that are of great neuropathological interest as they exhibit imaging alterations in the initial stages of AD development. The Pc is localized to the postero-medial portion of the parietal lobe and is involved in visuospatial imagery, episodic memory retrieval and self-consciousness [1]. The PCG is situated below and adjacent to the Pc and participates in internally direct thought, memory recollection, control of cognition and behavior modification due to environmental changes [2]. Moreover, these areas of the brain exhibit loss of gray matter in the early stages of AD as measured by magnetic resonance imaging (MRI) voxel-based morphometry [3]. Single photon emission computed tomography (SPECT) studies revealed that these brain regions have reduced cerebral blood flow in the initial stages of AD [4]. The Pc and PCG are watershed perfusion areas irrigated by the terminal branches of the pericallosal artery, a branch of the anterior cerebral artery, and/or by the terminal branches of the posterior cerebral artery [5]. Continuous arterial spin labeling (ASL) perfusion MRI studies have detected decreased perfusion in the Pc and PCG in mild cognitive impairment and AD relative to age-matched healthy controls [6]–[11]. A reduction in brain perfusion may precipitate the early failure of energy metabolism and consequential neuronal and glia injury observed in AD. Both Pc and PCG regions also demonstrate early stage hypometabolic activity as shown by fluorodeoxyglucose (FDG)-positron emission tomography (PET) scans, which is accentuated in apolipoprotein E (*APOE*) *ε4* gene allele carriers [12]–[20]. In addition, the PCG demonstrates a significant reduction of mitochondrial cytochrome C oxidase activity in young adults that carry the *APOE ε4* allele [21]. The Pc and PCG have been associated with a higher burden of fibrillar Aβ in cognitively normal older individuals as determined by Pittsburgh compound B (PiB)-PET scans [22], although this observation has been disputed [23]. Lastly, the Pc and PCG are components of the default mode network (DMN), a complex system of functionally linked neurons active when the individual is disconnected from the outside environment. The DMN has been suggested to malfunction in AD and other neurological disorders [24]–[26]. Whether these morphological, biochemical and hemodynamically-related perturbations affecting DMN communication are a facet of primary AD pathogenesis or are a secondary process remains to be established.

Aging is the major risk factor for the most prevalent neurodegenerative disorders, making it of paramount importance to contrast progressive molecular changes consistent with preservation of cognitive function with those associated with neurological disease. This task is complicated by the fact that normal aging and neurodegenerative diseases have many convergent phenotypes such as mitochondrial dysfunction [27]–[31], protein accumulation [32]–[36], inflammation [33], [37]–[40] as well as variable degrees of blood-brain barrier dysfunction [41]–[43]. It has been reported that only about 17% of elderly non-demented individuals demonstrate little or no evidence of brain degeneration [44]. To evaluate the regional neurochemical evolution of the Pc and PCG with aging, we quantified a selected group of proteins related to neurodegenerative diseases. Our objective was to investigate if along the process of putatively ‘normal brain aging’ the levels of these key molecules increase, decrease or remain stable. For this purpose, we examined individuals without clinical evidence of neurological disease or significant neurodegenerative disease changes and differentiated solely according to age: young adult (YA), middle-aged (MA) and oldest-old (OO). The OO group served as a biochemical benchmark of ‘successful aging’ without clinically detectable cognitive failure. A substantial body of evidence suggests that amyloid plaques form well in advance of the clinical manifestations of cognitive failure [45]. However, it is unclear whether such deposits induce immediate and directly proportionate consequential biochemical alterations or if responses are minimal until total amyloid deposition reaches a threshold level. Our experiments enabled us to assess if the biochemical alterations associated with dementia accumulate steadily with advancing age as well as to compare the molecular response profiles exhibited by YA and MA groups to that of neurologically successful aging present in the OO cohort. In addition, we discuss the relevance of these molecules in terms of normal function and their potential involvement in pathological processes.

## Materials and Methods

### Human subjects

Three cohorts without clinical evidence of neurological disease were selected and divided by age: 1) YA (range = 18–38 years), 2) MA (range = 53–65 years) and 3) OO (range = 91–99 years). The initial factors for inclusion were: the absence of amyloid deposits as well as the absence of clinically evident neurological disorders which was substantiated by neuropathological analyses **(** **Table 1** **and** **2** **)**. A limitation in the study is the unavoidable lack of clinical and some neuropathological information in the YA group, since these were young individuals who died as the result of unexpected accidents. For cases aged 38 and older, all subjects were participants in the Arizona Study of Aging and Neurodegenerative Disorders (AZSAND), a longitudinal clinicopathological study. Autopsies were performed by the Banner Sun Health Research Institute (BSHRI) Brain and Body Donation Program [46]. Written informed consent was obtained for all clinical and autopsy procedures, including those related to this study, and all approvals were obtained by the Banner Health and Western Institutional Review Boards. Eight of the YA brains (range 18–35 years) were obtained from The National Institute for Child Health and Human Development (NICHD) Brain and Tissue Bank for Developmental Disorders at the University of Maryland (Baltimore, MD) which receives cases from the Office of the Chief Medical Examiner (Baltimore, MD). Verbal informed consent was provided by the donor’s next of kin. The consent process is the same for minors and adults with the next of kin (an adult) being the person authorized to make decisions pertaining to the deceased. Verbal consent must be obtained during the two hour period between rounds at the Office of the Chief Medical Examiner and the time that the autopsy starts. One project coordinator reads the consent to the family and a witness then speaks to the family to verify that they have given consent for tissue donation. Once verbal consent is acquired, it is inappropriate to obtain a follow up written consent, however a follow up letter is sent to the families acknowledging their tissue donation. The verbal consent has been approved by the Internal Review Board of the Department of Health and Mental Hygiene for the State of Maryland and by the Internal Review Board for the University of Maryland School of medicine since 1991. Both Pc and PCG were available for all YA cases with the exception of case #2, for which PCG brain tissue was not available. All YA individuals died from multiple traumatic injuries **(** **Table 1** **)**. Case #9 (38 years old) was added to this group which originated from BSHRI. The second and third cohorts with average ages of 59 (range 53 to 65) and 93 (range 91 to 99) years were respectively classified as MA and OO. The last MMSE scores for all the OO individuals were 29–30. *APOE* genotypes for all cases were obtained from DNA isolated from cerebellar samples by a technique modified from Hixson and Vernier [47]. *APOE ε4* allele frequencies among the groups were: YA = 5.6%, MA = 0% and OO = 8.3% **(** **Table 1** **and** **2** **)**. Patient demographics (age, gender, postmortem interval (PMI)), brain weight, neuropathology scores (when available) and cause of death of the study subjects are given in **Table 1** **and** **2**.

**Table 1:** Study Subject Demographics and Neuropathological Scoring for Young Adults.

Young Adult(UofM)	Expiredage (yrs)	*APOE*	Gender	PMI(hrs)	Brainweight (g)	Presence ofamyloid	AT8 postivetau	Cause of Death			
1	18	3/3	M	9	ND	0	0	Motor vehicle accident			
2	19	3/3	F	7	ND	0	0	Motor vehicle accident			
3	20	3/3	M	6	ND	0	+	Unspecified fatal traumatic injuries			
4	22	3/3	M	9	ND	+	0	Motor vehicle accident			
5	22	2/3	M	8	ND	0	+	Motor vehicle accident			
6	24	¾	M	8	ND	0	0	Motor vehicle accident			
7	32	3/3	F	12	ND	0	+	Pedestrian vehicle fatality			
8	35	3/3	M	12	ND	0	0	Motor vehicle accident			
**Young Adult** **(BSHRI)**	**Expired** **age (yrs)**	***APOE***	**Gender**	**PMI (hrs)**	**Brain** **weight (g)**	**Total** **plaque score**	**Total NFT** **score**	**Braak score**	**WMR total**	**CAA score**	**Cause of** **Death**
9	38	3/3	M	3	1350	0	I	I	0	0	Esophageal cancer
**Mean**	**26**			**8**							

**Table 2:** Study Subject Demographics and Neuropathological Scoring for Middle-Aged and Oldest-Old.

Middle-Aged (BSHRI)	Expiredage(yrs)	*APOE*	Gender	PMI(hrs)	Brainweight (g)	TotaPlaquescore	TotalNFTscore	Braakscore	WMRtotal	CAAscore	Cause of Death
20	53	3/3	M	4	1456	0	1.0	I	0	0	Liver cancer
21	58	2/3	F	3	1404	0	2.0	I	0	0	Non-Hodgkin’s lymphoma
22	59	3/3	F	3	1162	1.5	0.5	I	6	0	Suprarenal cancer
23	61	3/3	M	2	1220	0.5	0.5	I	2	5	Lung cancer
24	65	3/3	M	4	1400	0	1.0	I	2	0	Lung cancer
**Mean**	**59**			**3**	**1328**	**0.4**	**1.0**		**2.0**	**1**	
**Oldest-old (BSHRI)**	**Expired age (yrs)**	***APOE***	**Gender**	**PMI (hrs)**	**Brain weight (g)**	**Total plaque score**	**Total NFT** **score**	**Braak** **score**	**WMR** **total**	**CAA score**	**Cause of Death**
30	91	2/3	F	5	1112	0	5.0	III	2	0	Cardiorespiratory arrest
31	91	3/4	M	2	1330	0	7.5	IV	1	0	Renal failure
32	91	2/3	F	3	1100	0	6.5	IV	1	0	COPD
33	91	3/3	F	7	1100	0	4.0	IV	4	0	Congestive heart failure
34	92	3/3	M	3	1225	0	8.5	IV	1	0	Renal failure
35	99	3/3	F	4	975	0	3.5	III	5	0	Congestive heart failure
**Mean**	**93**			**4**	**1140**	**0.0**	**5.8**		**2.3**	**0**	

UofM, University of Maryland; BSHRI, Banner Sun Health Research Institute; yrs, years; APOE, apolipoprotein E genotype; PMI, postmortem interval; hrs, hours; g, grams; NFT, neurofibrillary tangles, WMR, white matter rarefaction; F, female; M, male; ND = not determined; COPD, chronic obstructive pulmonary disease.

### Neuropathological evaluation

#### BSHRI cases

Forty µm brain sections were used for histological studies. Campbell-Switzer, Thioflavine-S, Gallyas and hematoxylin and eosin (H&E) staining procedures were used to identify and score the severity of amyloid plaques, neurofibrillary tangles (NFT), Lewy-type synucleinopathy and white matter rarefaction [46]. Amyloid plaque densities were rated as none, sparse, moderate and frequent and reported numerically as 0, 1, 2 and 3, respectively, using the CERAD templates [48], [49]. The Braak stage (I–VI) was determined by the previously described method by Braak and Braak [50]. In addition, total plaque score was obtained by summation of all amyloid plaque types (compact, neuritic, classical and diffuse) in 5 brain regions: frontal, temporal, parietal, hippocampal and entorhinal, for a total maximum score of 15. Similarly, the total NFT score was evaluated as described for the total plaque score, using the published CERAD templates. White matter rarefaction (WMR) and total cerebral amyloid angiopathy (CAA) scores were ascertained in the frontal, temporal, parietal and occipital lobes and scored as none, mild, moderate and severe which were converted into numeric scores of 0, 1, 2, 3 (maximum total score of 12). Lewy body disease staging followed the Unified scheme [51].

### Neuropathological evaluation

#### University of Maryland Cases

Brain sections from the Pc and PCG were cut at 40 µm and stained for Aβ using the 6E10 clone antibody (Millipore, Billerica, MA), for phosphorylated tau using AT8 antibody (Millipore) [52], and were also processed with Campbell-Switzer silver stain and thioflavine-S to assess the presence of AD neuropathology [46].

### ELISA quantification

Gray matter (200 mg) from the Pc and PCG were submitted to ELISA. In brief, brain samples for amyloid-β (Aβ)1–40, Aβ1–42, tau and α-synuclein ELISAs were homogenized in 90% glass distilled formic acid (GDFA) and centrifuged at 250,000 x *g* for 1 hr. The supernatant was dialyzed against distilled water followed by 50 mM ammonium bicarbonate. The dialyzed specimens were lyophilized and reconstituted in 5 M guanidine hydrochloride (GHCl), 50 mM Tris-HCl, pH 8.0, containing a protease inhibitor cocktail (PIC, Roche Diagnostics, Mannheim, Germany). For complete sample preparation details, see reference [53]. Brain specimens used in ELISA experiments to quantify ApoE, tumor necrosis factor-α (TNF-α), CD200, brain-derived neurotrophic factor (BDNF) and glial fibrillary acidic protein (GFAP) were homogenized in RIPA buffer (see below).

Total protein was quantified in all samples with the Pierce Micro BCA Protein Assay kit. ELISA kits from Life Technologies Corp. (Carlsbad, CA) were used to quantify Aβ, tau and α-synuclein while TNF-α and BDNF levels were measured with a kit from PromoKine (Heidelberg, Germany), according the manufacturers’ instructions. The detailed ELISA protocols for ApoE, CD200 and GFAP are published elsewhere [53].

### Western blot analysis

Gray matter (200 mg) from the Pc and PCG was homogenized in 2 ml RIPA buffer (Sigma, St. Louis, MO) containing PIC (Roche) using an Omni TH tissue grinder (Kennesaw, GA) and centrifuged at 14,000 x *g* for 20 min in a Beckman 22R centrifuge. Total protein was determined in the recovered supernatant with a Micro BCA protein assay (Pierce, Rockford, IL). Supernatant samples, adjusted to contain equivalent total protein quantities, were placed into 2X XT sample buffer (Bio-Rad, Hercules, CA) supplemented with 50 mM dithiothreitol and heated for 10 min at 80°C. Proteins were separated on Criterion XT 4–12% Bis-Tris 26-well gels (Bio-Rad) with XT 1XMES running buffer (Bio-Rad) containing NuPage antioxidant (Life Technology Corp.) and transferred onto 0.45 µm nitrocellulose membranes (Life Technology Corp.). Certain antibodies (CT20APP, APLP1) required the nitrocellulose membranes to be boiled in 1XPBS (EMD Chemicals, Gibbstown, NJ) for 5 min following protein blotting to expose protein epitopes. Furthermore, APLP2 was run under non-reducing conditions. Membranes were blocked in 5% Quick-Blocker (G-Biosciences, St. Louis, MO) in 1XPBS containing 0.5% Tween 20. Primary and secondary antibodies **(** **Table 3** **)** were diluted in blocking buffer. After protein detection (SuperSignal WestPico Chemiluminescent Substrate, Pierce) and visualization (CL-Xpose film (Pierce) and Konica-Minolta SRX-101A autoprocessor, Wayne, NJ), antibodies were stripped from the membranes with Restore Western Blot Stripping Buffer (Pierce) and re-probed with anti-mouse, anti-rabbit actin or GAPDH antibody **(** **Table 3** **)** to provide a protein loading control. The trace quantity feature in Quantity One software (Bio-Rad) is defined as the measured area under each band’s intensity profile curve and was used to determine the optical density (OD) x mm. The trace quantity of the primary proteins was divided by the trace quantity of the actin or GAPDH re-probes. This adjusted value was used for statistical analyses.

**Table 3:** Antibodies Used for Western Blot Analysis.

Primary antibody	Antigen specificity or immunogen	Secondary antibody	Company/Catalog #
APP573	aa 573–596 of APP	M	Covance/SIG-39180
CT20APP	Last 20 aa of APP	R	Covance/SIG-39152
BACE1	3D5 clone, aa 46–460	M	Kindly provided by Dr. R. Vassar
APLP1	Mouse myeloma cell line NS0-derived recombinant human APLP1	M	R&D Systems/MAB3908
APLP2	Mouse myeloma cell line NS0-derived recombinant human APLP2	M	R&D Systems/MAB49451
ApoJ	Recombinant ApoJ	G	Millipore/AB825
PEDF	Human PEDF	R	BioProducts MD/AB-PEDF1
S100B	Synthetic C-terminal peptide of human S100B	R	Abcam/ab52642
Actin Ab-5	Clone C4	M	BD Transduction Laboratories/A65020
Actin	N-terminus of human α-actin	R	Abcam/Ab37063
GAPDH	Full-length human GAPDH protein	M	Life Technologies/39-8600

APP, amyloid-β precursor protein; aa, amino acids; BACE, β-site APP cleaving enzyme; APLP, amyloid precursor-like protein; BACE, β-site APP-cleaving enzyme; Apo, apolipoprotein; PEDF, pigment epithelium derived factor; ApoE, apolipoprotein E; ApoJ, apolipoprotein J; M, HRP conjugated AffiniPure goat-anti mouse IgG (catalog #115-035-146, Jackson Laboratory); R, HRP conjugated AffiniPure goat-anti rabbit IgG, (catalog #111-035-144 Jackson Laboratory); G, HRP conjugated AffiniPure bovine-anti goat IgG (catalog #805-035-180).

### Statistical analysis

All data were analyzed with GraphPad Prism 5 software (La Jolla, CA). Significance was set at *p*≤0.05. Given the small sample size, the 3 age groups were statistically compared using the non-parametric Kruskall-Wallis test. Dunn’s multiple comparison test was used to adjust for multiple group comparisons and yielded the following p-value ranges for groupwise comparisons that were statistically significant: *p = 0.05–0.01; **p = 0.01–0.001; ***p≤0.001. We are well aware of the small number of individuals in the cohorts under study. This is due to the difficulty in obtaining high quality tissues with short postmortem intervals and without evidence of neuropathological signs of AD or other associated neurodegenerative disorders.

## Results

### I. Human Subjects and Neuropathology Analyses

Whole brain samples were not available for cases 1–8 in the YA cohort, so it was not possible to perform standard neuropathological examinations such as total plaque score, Braak staging, total NFT score, etc. However, assessments of Aβ load in the Pc and PCG were determined using immunohistochemistry (6E10 antibody), Campbell-Switzer and thioflavine-S staining. None of the YA cases exhibited any amyloid plaque neuropathology with the sole exception of case #4 which harbored some vascular/parenchymal amyloid cores. Phosphorylation of tau was determined using AT8 antibody immunohistochemistry. Cases #3, #5 and #7 had rare positive tau fibers while the remaining YA cases did not possess detectable phosphorylated tau-related pathology. Case #9, who was 38 years old and the only YA case originating from BSHRI, as well as all cases in the MA and OO groups (all from BSHRI) had total plaque scores of zero or low values **(** **Table 1** **and** **2** **)**. Total NFT scores were ≤2 (out of a max. of 15) in the MA group, while these values were intermediate in the OO nonagenarian cohort (range 4.0–8.5; out of a max. of 15). The Braak score was I for the YA case #9. All individuals in the MA group had a Braak Score of I while the OO cohort ranged from III–IV **(** **Table 2** **)**. The WMR score was 0 for YA case #9 and the MA group and had an average of 2.0 (out of a max. of 12). The average WMR score in the OO group was 2.3 **(** **Table 2** **)**. The only case to have detectable cerebral amyloid angiopathy (CAA) was case #23 in the MA group **(** **Table 2** **)**. Lastly, the Lewy body stage was 0 in all the BSHRI participants in the study.

### II. APP/CTF/Aβ and related peptides

**Figure 1:**
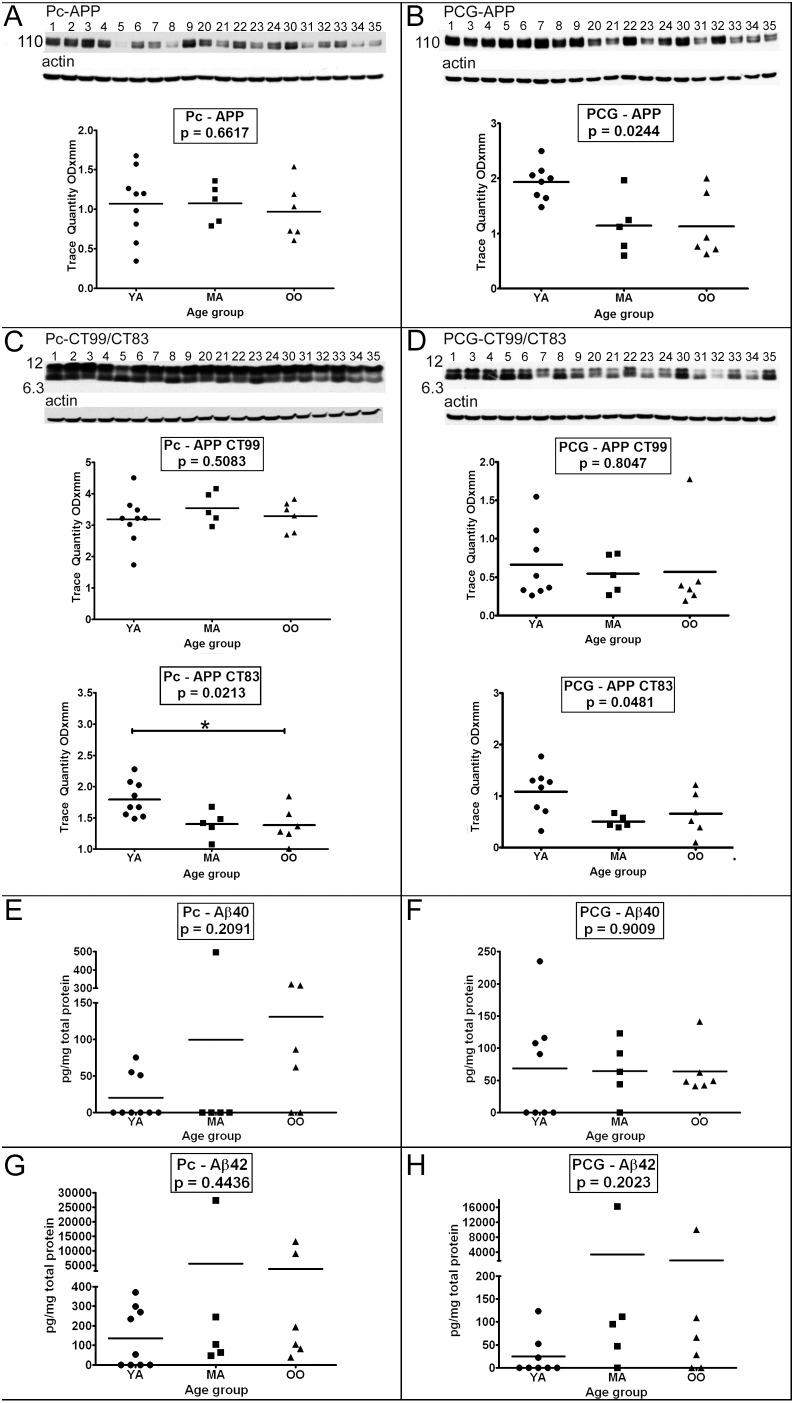
Amyloid precursor protein and proteolytic-derived peptides assessed by Western blot or ELISA. Sample numbers correspond to young adult (1–9), middle-aged (20–24) and oldest-old (30–35). Full length APP was detected in the precuneus (A) and posterior cingulate gyrus (B) by Western blot. C-terminal peptides (CT99 and CT83) of APP were determined by Western blot using an antibody against the last 20 amino acids of APP in the precuneus (C) and posterior cingulate gyrus (D). A total of 40 µg of total protein was loaded per lane. Data are reported in optical density units and were adjusted for actin. Actin loading probes are shown below each primary antibody blot. The molecular weight is shown on the left side of each blot. Brain tissue that was homogenized in GDFA/GHCl (see Materials and Methods) was used to quantify Aβ40 and Aβ42 in the precuneus (E, G) and posterior cingulate gyrus (F, H) by ELISA. The ELISA concentrations are reported in pg per mg of total protein. Abbreviations: Pc, precuneus; PCG, posterior cingulate gyrus; YA, young adult; MA, middle-aged; OO, oldest-old; APP, amyloid precursor protein; CT, C-terminal. The 3 age groups were statistically compared using the non-parametric Kruskall-Wallis test followed by the Dunn’s multiple comparison test (*p = 0.05–0.01; **p = 0.01–0.001; ***p≤0.001).

The mean levels of amyloid precursor protein (APP) did not significantly change with age in the Pc **(** **Figures 1A** **)**. However, the YA PCG levels of APP were significantly higher than in the MA and OO groups (**Figure 1B**, *p* = 0.0244). Interestingly, cases #5 and #8 in the YA Pc and #31, #34 and #35 in the OO Pc had a relatively low abundance of APP **(** **Figure 1A** **)**. In the PC and PCG, the levels of the APP C-terminal fragment (CTF), CT99, did not significantly differ among the 3 age groups while in both the Pc and PCG, CT83 significantly decreased with age (**Figure 1C**, *p* = 0.0213 and **Figure 1D**, *p* = 0.0481, respectively). However, the levels of Aβ40 and Aβ42 did not exhibit significant changes among the 3 age groups and there was a high degree of individual variability in the Pc and PCG **(** **Figures 1E–1H** **)**. Several cases had relatively high Pc Aβ40, Pc Aβ42 and PCG Aβ42 levels (**Figure 1E** – case #23; **1G** – case #23, #30, #33; **1H** – case #23, #33). Case #23 was the only individual to have evidence of CAA **(** **Table 2** **)** which may explain the relatively high amounts of Aβ in this subject. Excluding these cases from the statistical calculations also yielded non-significant *p*-values **(** **Figure 1E**, p = 0.06; **Figure 1G** p = 0.45; **Figure 1H** p = 0.42**)**. Overall, the majority of cases had Aβ40 and Aβ42 concentrations in the pg/mg total protein range **(** **Figures 1E–1H** **)**, although in 29 out of 78 ELISA determinations for the Aβ peptides, the values were below the limit of detection **(** **Figures 1E–H** **)**.

### III. BACE1 and APLPs

**Figure 2:**
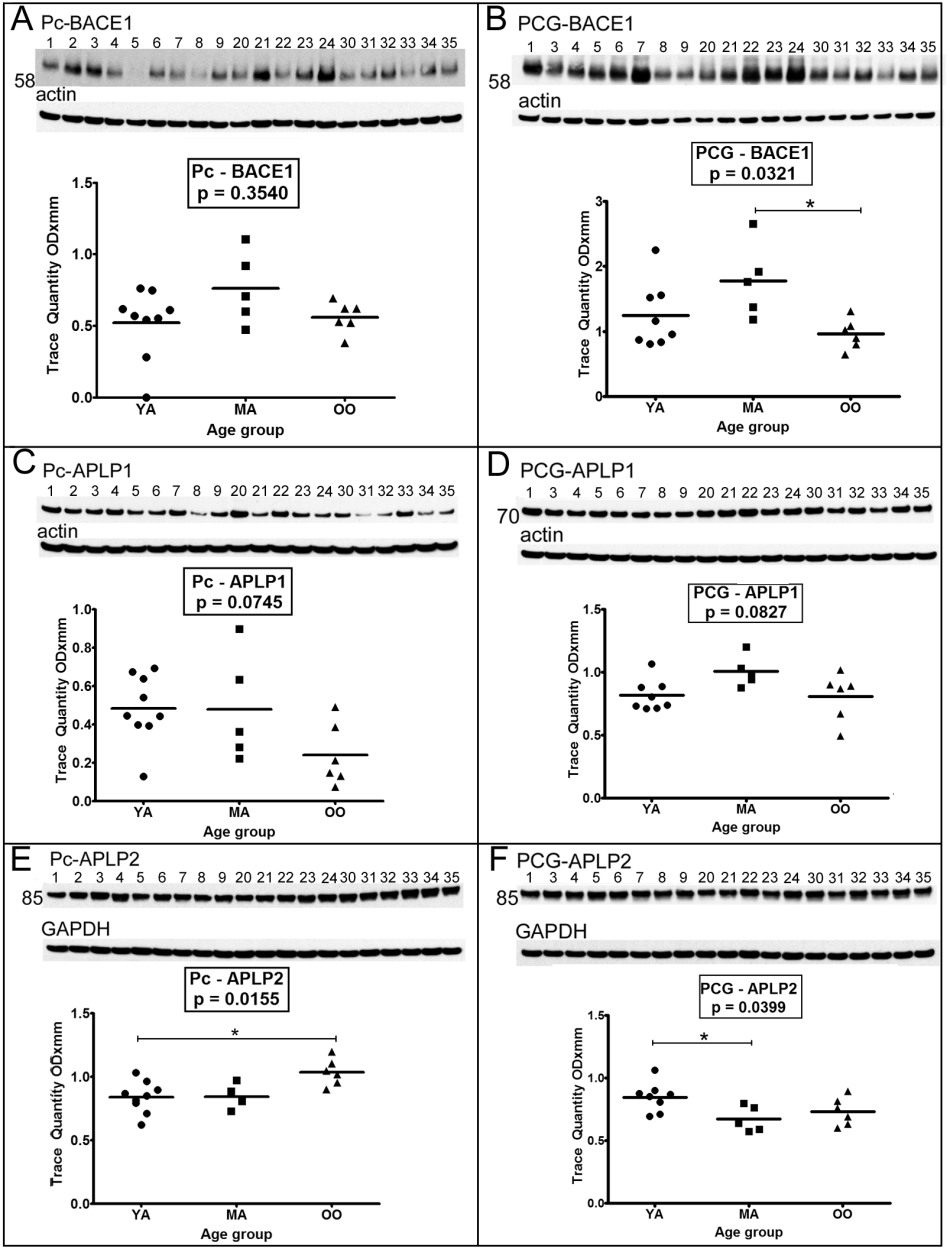
Western blot analyses of BACE1 and APLP1 and APLP2. Sample numbers correspond to young adult (1–9), middle-aged (20–24) and oldest-old (30–35). Western blotting was used to detect BACE1 in the precuneus (A) and posterior cingulate gyrus (B), APLP1 in the precuneus (C) and posterior cingulate gyrus (D) and APLP2 in the precuneus (E) and posterior cingulate gyrus (F). All Western blots were loaded with a total of 40 µg of protein per lane. APLP2 blots were performed under non-reducing conditions. Data are reported in optical density units and were adjusted for actin (BACE1, APLP1) or GAPDH (APLP2). Actin and GAPDH loading probes are shown below each primary antibody blot. Abbreviations: Pc, precuneus; PCG, posterior cingulate gyrus; YA, young adult; MA, middle-aged; OO, oldest-old; BACE1, β-site amyloid precursor protein-cleaving enzyme-1; APLP, amyloid precursor-like protein. For statistical treatment see legend to Figure 1.

The mean β-site APP-cleaving enzyme (BACE)1 levels exhibited an interesting trend in both the Pc and PCG. Enzyme levels increased in the MA in relation to the YA, but decreased in the OO to amounts similar to those in the YA **(** **Figures 2A and 2B** **)**. The differences did not reach statistical significance in the Pc (**Figure 2A**, *p* = 0.3540). However, in the PCG, the MA had significantly more BACE1 than the OO group (**Figure 2B**, *p* = 0.0321; Dunn’s multiple comparison test *p* = 0.05–0.01). The Pc cases #5 and #8 demonstrated low BACE1 quantities **(** **Figures 2A** **)** which were proportionate to those observed for the APP **(** **Figures 1A** **)**.

The amounts of amyloid precursor-like protein (APLP)1 detectable in the Pc and PCG did not show significant differences between age cohorts **(** **Figures 2C and 2D** **)**. In contrast, differences in APLP2 levels in the Pc and PCG reached statistical significance, but these 2 different areas of the brain showed different trends (**Figure 2E** *p* = 0.0155 and **Figure 2F** *p* = 0.0399). In the Pc, APLP2 levels in the YA were significantly lower than in the OO subjects (**Figure 2E**, *p* = 0.0155; Dunn’s multiple comparison test *p* = 0.05–0.01). In the PCG, APLP2 levels in the YA were significantly higher than in the MA subjects (**Figure 2E**, *p* = 0.0399; Dunn’s multiple comparison test *p* = 0.05–0.01).

### IV. Tau and α-synuclein

**Figure 3:**
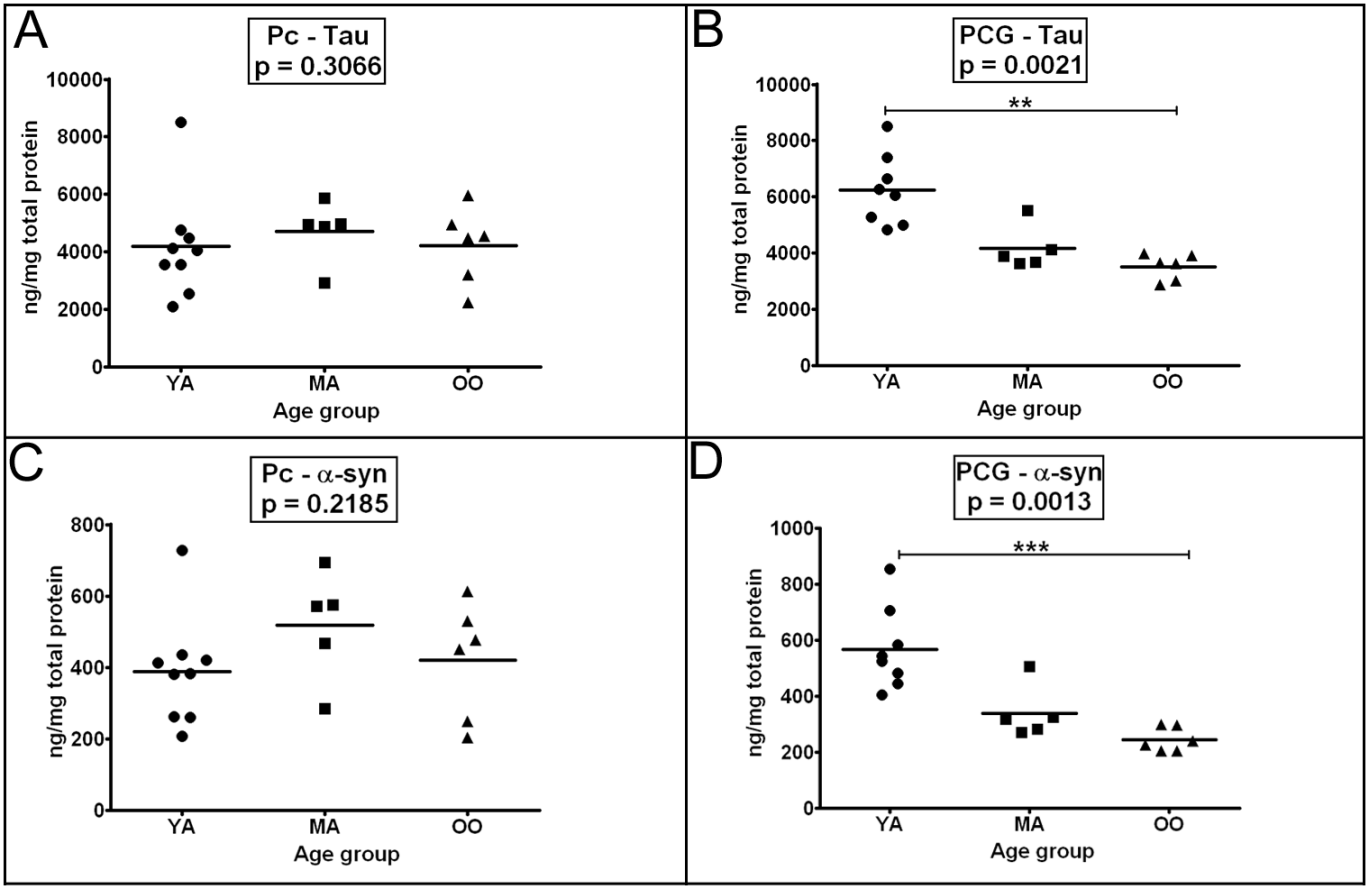
ELISA quantitative analyses of tau and α-synuclein. Sample numbers correspond to young adult (1–9), middle-aged (20–24) and oldest-old (30–35). Tau results are shown in Figures A and B and α-synuclein are presented in Figures C and D for both precuneus and posterior cingulate gyrus as indicated. Concentrations are reported in ng per mg of total protein. Tau and α-synuclein were detected in GDFA/GHCl homogenates (for details see Materials and Methods section). Abbreviations: Pc, precuneus; PCG, posterior cingulate gyrus; YA, young adult; MA, middle-aged; OO, oldest-old; α-syn, α-synuclein. For statistical analyses see legend to Figure 1.

The mean levels of tau and α-synuclein remained fairly consistent among the 3 cohorts in the Pc **(** **Figures 3A and 3C** **)**. In contrast, in the PCG, both tau and α-synuclein levels significantly decreased with age (*p* = 0.0021 and *p* = 0.0013, respectively) among the 3 groups under study, with greater amounts in the YA, intermediate in the MA and lesser in the OO cohorts **(** **Figures 3B and 3D** **)**. Furthermore, a comparison between YA and OO groups also yielded significant differences (**Figure 3B** **,** tau *p* = 0.01–0.001 and **Figure 3D** **,** α-synuclein *p*≤0.001).

### V. Apolipoproteins

**Figure 4:**
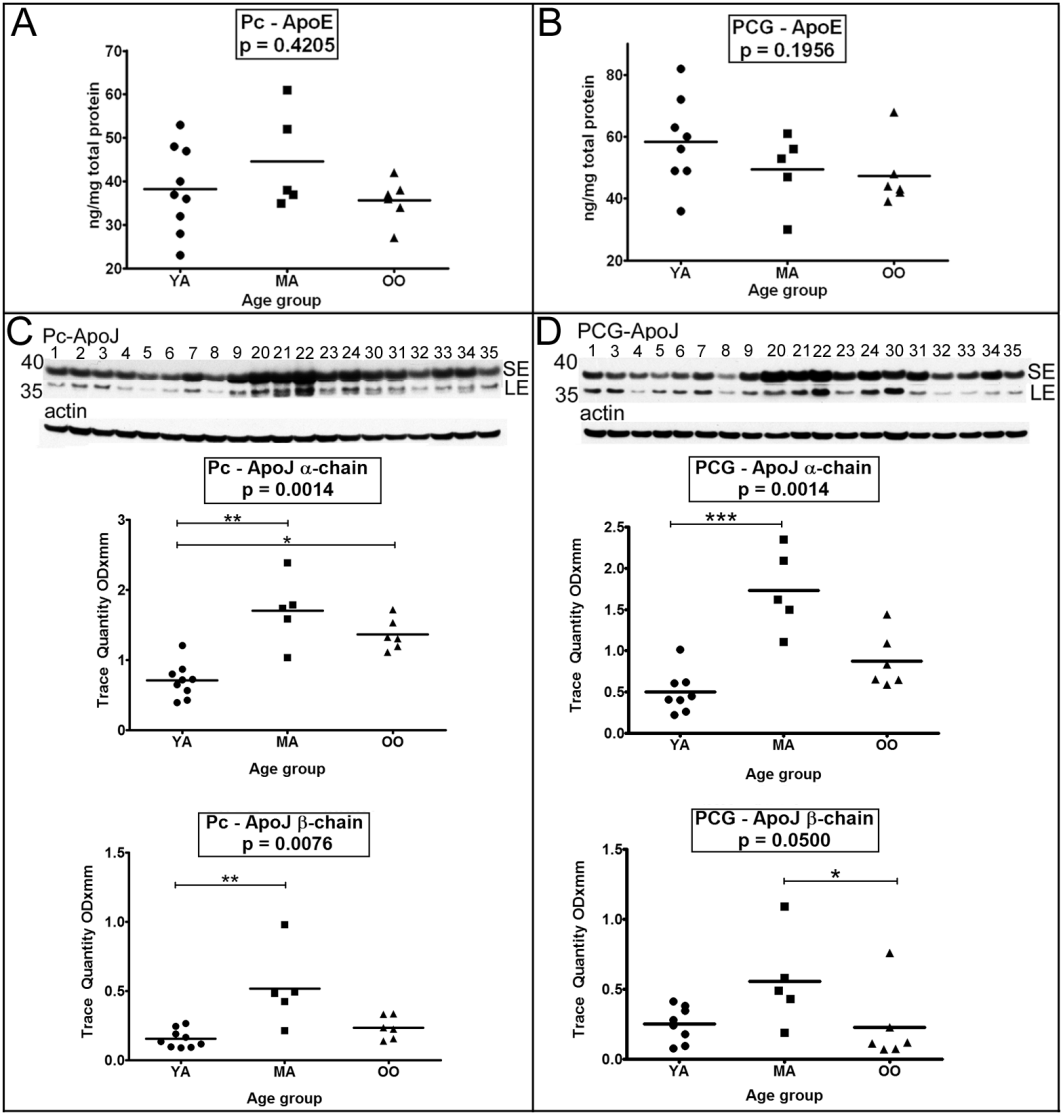
ELISA quantitative analyses of total ApoE and Western blot analyses of ApoJ. Sample numbers correspond to young adult (1–9), middle-aged (20–24) and oldest-old (30–35). ELISA analyses for ApoE were performed in RIPA homogenates from the precuneus (A) and posterior cingulate gyrus (B). ELISA concentrations are reported in ng per mg of total protein. Western blotting was used to visualize ApoJ-α and ApoJ-β chains in the precuneus and posterior cingulate gyrus (C, D). For Western blots, a total of 40 µg of total protein was loaded per lane. Data are reported in optical density units and were adjusted for actin. Actin loading probes are shown below each primary antibody blot. The molecular weight is shown on the left side of each blot. Abbreviations: Pc, precuneus; PCG, posterior cingulate gyrus; YA, young adult; MA, middle-aged; OO, oldest-old; ApoE, apolipoprotein E; ApoJ, apolipoprotein J. For statistical treatment see legend to Figure 1.

ApoE **(** **Figures 4A and 4B** **)** and ApoJ (clusterin) **(** **Figures 4C and 4D** **)** were all analyzed in both the Pc and PCG. Only ApoJ showed statistically significant differences. The α-chain of this protein sharply increased from YA to MA in both the Pc (**Figure 4C**, *p* = 0.01–0.001) and PCG (**Figure 4D**, *p*≤0.001). The α-chain of ApoJ was also significantly increased in the OO relative to YA in the Pc (**Figure 4C**, *p* = 0.05–0.01). In addition, the Pc ApoJ β-chain level was also significantly elevated in the MA group in comparison to the YA cases **(** **Figures 4C**, *p* = 0.01–0.001**)**. ApoJ β-chain levels in the PCG followed a similar age-related pattern (**Figure 4D**, *p* = 0.05; Dunn’s multiple comparison p = 0.05–0.01).

### VI. Inflammatory and Vascular Proteins

**Figure 5:**
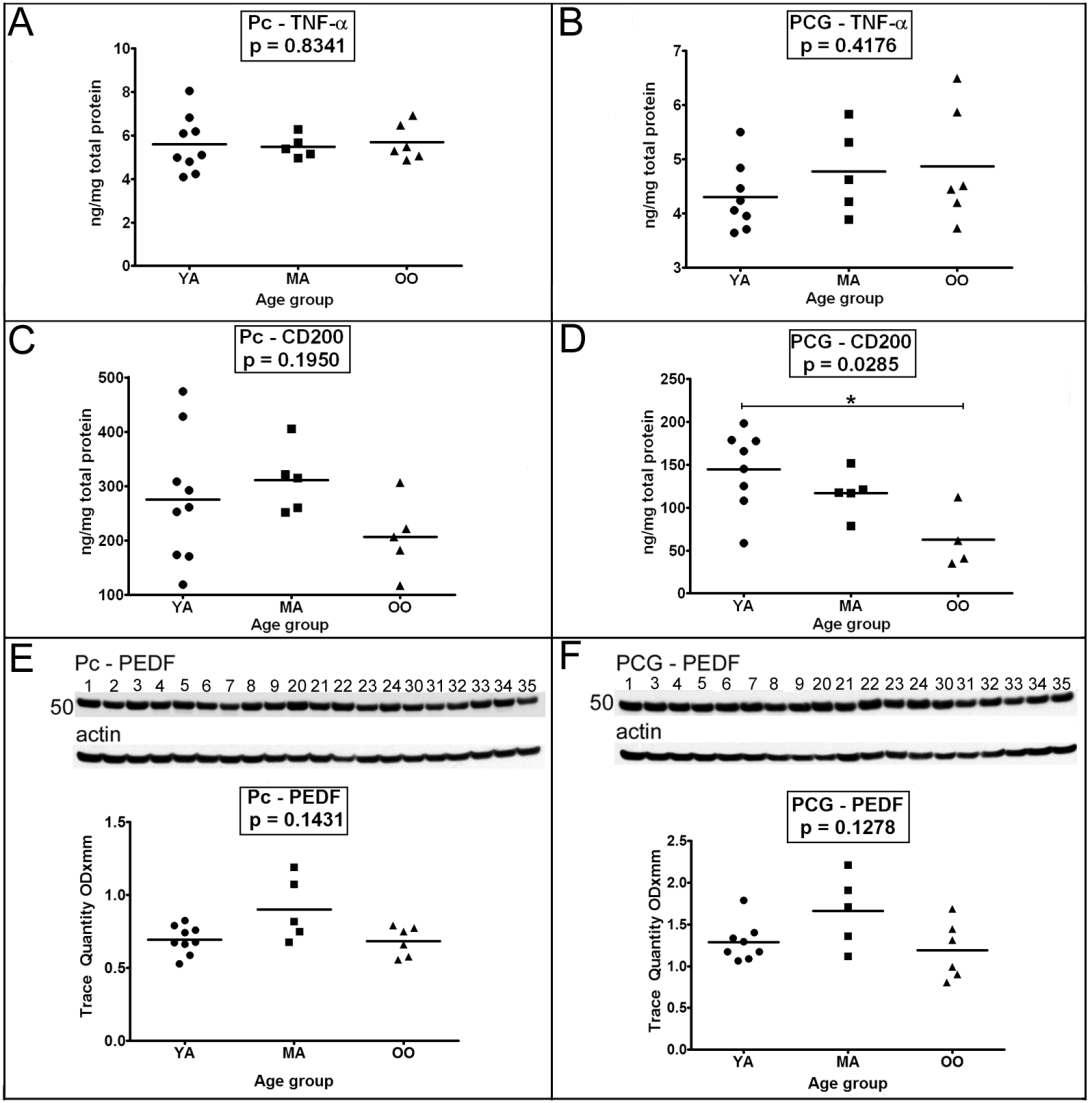
ELISA quantitative analyses of TNF-α and CD200 and Western blot analyses of PEDF. As indicated in the Figure both the precuneus (A, C and E) and posterior cingulate gyrus (B, D and F) were investigated. Sample numbers, shown above each blot, correspond to young adult (1–9), middle-aged (20–24) and oldest-old (30–35). ELISA concentrations are reported in ng per mg of total protein. For Western blot analyses a total of 40 µg of total protein was loaded per lane. Data are reported in optical density units and were adjusted for actin. The actin loading probes is shown below the primary antibody blot. The molecular weight is shown on the left side of each blot. Abbreviations: Pc, precuneus; PCG, posterior cingulate gyrus; YA, young adult; MA, middle-aged; OO, oldest-old; TNF-α, tumor necrosis factor-α; PEDF, pigment epithelium-derived factor. For statistical treatment see legend to Figure 1.

The pro-inflammatory molecule tumor necrosis factor (TNF)-α, on the average, demonstrated very small elevations with increasing age in the PCG **(** **Figure 5B** **)** while there were no changes in the average levels of TNF-α in the Pc **(** **Figure 5A** **)**. On the other hand, the anti-inflammatory protein, CD200, declined with age (*p* = 0.0285) and was significantly decreased in the OO relative to the YA cases in the PCG **(** **Figure 5D** **.** *p* = 0.05–0.01**)**. However, this trend was not observed in the Pc **(** **Figure 5C** **)**. The relative mean quantities of the anti-angiogenic factor pigment epithelium derived factor (PEDF) were similar in YA and OO cohorts and slightly elevated in MA subjects in both the Pc (*p* = 0.1431) and PCG (*p* = 0.1278); however the values were not statistically significant (**Figure 5E and 5F**, respectively).

### VII. BDNF, GFAP and S100B

**Figure 6:**
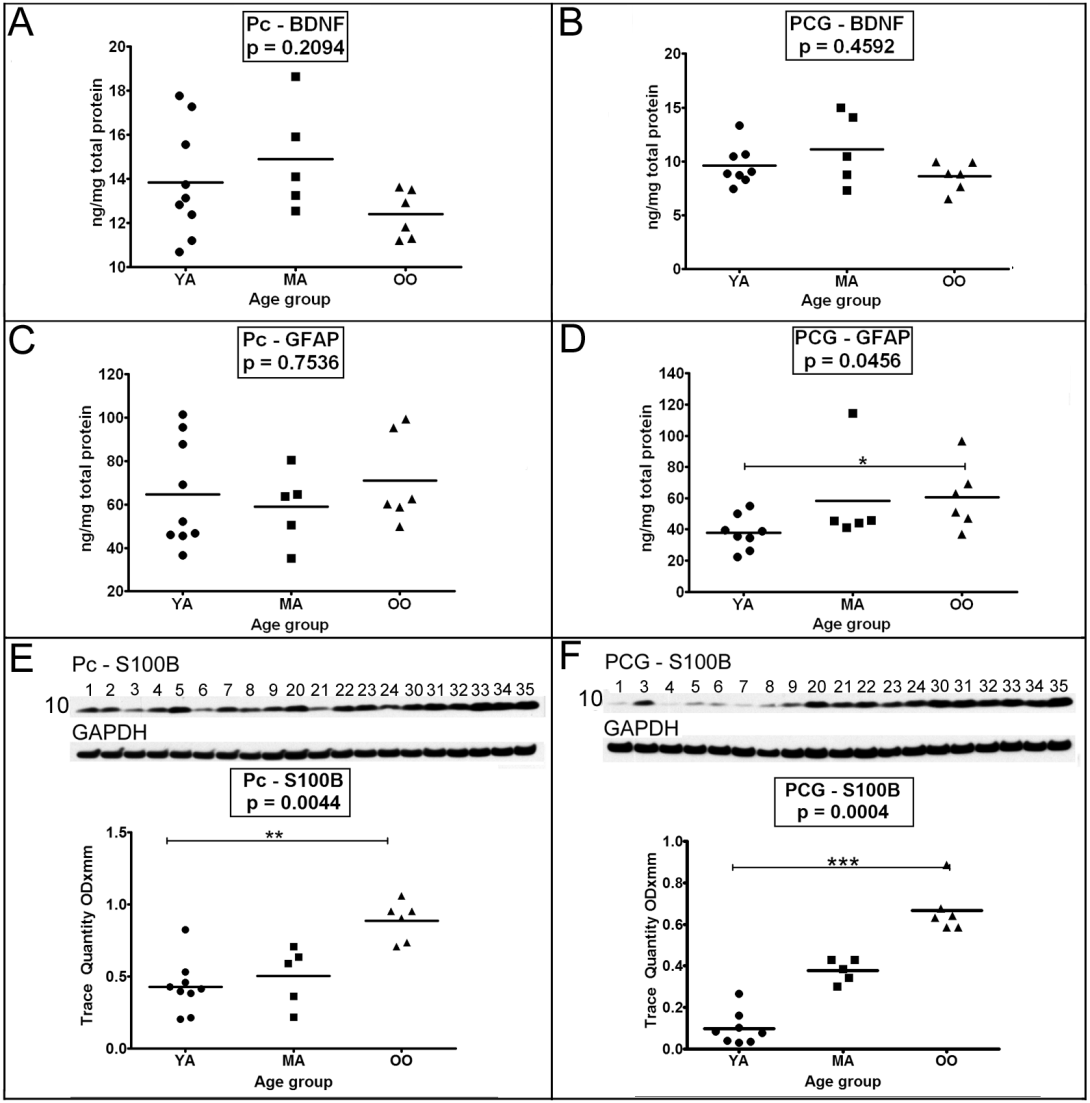
ELISA quantitative analyses of BDNF and GFAP and Western blot analyses of S100B. As indicated in the figure both the precuneus (A, C and E) and posterior cingulate gyrus (B, D and F) were investigated. Sample numbers, shown above each Western blot correspond to young adult (1–9), middle-aged (20–24) and oldest-old (30–35). ELISA concentrations are reported in ng per mg of total protein. For the S100B Western blot, a total of 25 µg of total protein was loaded per lane. Data are reported in optical density units and were adjusted for GAPDH. The GAPDH loading probe is shown below the primary antibody blot. The molecular weights are shown on the left side of each blot. Abbreviations: Pc, precuneus; PCG, posterior cingulate gyrus; YA, young adult; MA, middle-aged; OO, oldest-old; BDNF, brain-derived neurotrophic factor; GFAP, glial fibrillary acidic protein; S100B, S100 calcium binding protein-B. For statistical analyses see legend to Figure 1.

While the mean levels of the neurotrophin BDNF tended to be lower in the OO group, the values were not statistically significant in either the Pc or PCG **(** **Figure 6A and 6B** **)**. GFAP levels exhibited no significant changes in the Pc **(** **Figure 6C** **)**, while in the PCG a significant increase with age was observed between the YA and OO groups (**Figure 6D**, *p* = 0.05–0.01). Interestingly, the neurotrophic factor S100B consistently and dramatically increased with age among the 3 groups under study, being statistically significant in both the Pc (**Figure 6E**, *p* = 0.0044) and PCG (**Figure 6F**, *p* = 0.0004). S100B levels were significantly increased in the OO cohort relative to the YA subjects in both the Pc (**Figure 6E**, *p* = 0.01–0.001) and PCG (**Figure 6F**, *p*≤0.001).

## Discussion

Our investigation focused on how proteins involved in neurodegeneration change with non-pathological aging in the Pc and PCG. The selection of these brain regions was based on the observation that these two regions of the brain have been considered to be initial sites in the development of AD [4], [17], [54]–[58]. Our goal was to investigate a series of variables which are classically associated with the development of AD and quantify them along the process of aging from YA to OO cohorts. The MA group died prematurely from other non-neurological, cancer-related morbidities and hence were short of reaching the present average life expectancy (∼80 years) prevailing in the USA. One striking observation of our study is the individual variability that occurred among the 20 subjects under investigation.

Our data suggest that the brains of individuals without any symptoms of neurological disorders, lose about 15% weight between the 6–7^th^ and 10^th^ decade of life (mean brain weight in the MA 1328 g vs.1140 g in OO; *p* = 0.035). Multiple studies have reported a loss of brain volume that occurs with age [32], [59]–[64]. Stereological studies showed that normal neuronal loss accounts for ∼10% between the ages of 20–90 years while there is no statistically significant loss of glial cells [65]. Alternatively, others have reported that there is no apparent loss in numbers of neurons in normal aging, but there is a loss of synapses and changes in the size and structure of neurons [31], [33], [66]–[68]. We suggest that the differences between MA and OO groups, which encompass 3 decades, could be adjudicated to general aging atrophy. However, in the absence of neuropathology, which may account for obvious neuronal and glial loss [69] and myelin decline [70], [71], cellular shrinkage, dehydration and diffuse wasting similar to that observed in skeletal muscle sarcopenia may account for the reduction in brain weight in the last decades of life.

Our data reveal that the burden of amyloid plaques can be substantially low or undetectable in MA individuals and in mentally healthy nonagenarians suggesting that the physiological mechanisms responsible for triggering amyloid deposition might be absent in some elderly individuals without clinical symptoms of dementia. Multiple studies have also shown that individuals with amyloid plaques can be non-demented [44], [53], [72]–[80] which undermines the idea of these lesions as being the chief culprit for the expression of dementia. In this respect, it has been recently suggested that at equal amyloid plaque loads the difference between demented and non-demented individuals lies upon the higher amount of Aβ oligomers in the former relative to the latter [81].

The incidence of NFT formation increases with age [73], [82]–[84], and the difference between demented and non-demented subjects is mainly due to greater tangle densities and the spread of tangles across the neocortex in AD [85]. Likewise, we found that the NFT score was very low in non-demented MA subjects which was matched by a Braak stage of I, while in the OO, without the symptoms of dementia and without amyloid deposits, the NFT scores reached an intermediate average value of 5.8 (out of a maximum score of 15) with Braak scores of III–IV. The NFT score was not used for the selection of study subjects. Taken together these observations suggest that in humans the presence of amyloid plaques does not precede or is necessary for the development of NFT. Limited NFT deposition is consistent with normal aging and is either tolerable or perhaps serving as an adaptive/rescue event to preserve cytoskeletal integrity and axonal transport patency or involved in the management of hazardous waste resulting from damaged organelles [86].

White matter rarefaction, a neuropathological entity that has been variously interpreted as resulting from white matter hypoperfusion or primary oligodendroglial pathology or as being secondary to cortical gray matter loss, leads to a significant loss of myelin and axonal degeneration in AD [87]–[89]. In the current study, the total WMR score was relatively low or absent in MA and OO individuals (averaging 2.0 and 2.3, respectively, out of a maximum score of 12, suggesting relative myelin and axonal conservation. In a previous study [53], we observed that in non-demented OO-high pathology control (HPC = individuals with high amyloid plaque burden, but without dementia) subjects, the total average WMR score was 2.3 while in OO cases with AD it was 5.5. Likewise, the average CAA score in OO-HPC cases was 3.5 which contrasted with the OO-AD score of 6.5 (out of a maximum score of 12). These two observations suggest that the differences between OO-AD on the one hand, and OO or OO-HPC on the other, may be due to a generally better preserved brain microcirculation in the latter, although relative preservation of oligodendroglia and cortical gray matter must also be considered. Supporting the changes in microcirculation, hemodynamic assessments using transcranial Doppler ultrasound demonstrated significantly altered measures in AD, in terms of mean flow velocities and pulsatility indices, when compared to non-demented control subjects [90]. Along this topic, degenerated string capillaries were elevated in OO-AD, implying greater microvascular dysfunction, when compared to septuagenarian and nonagenarian non-demented groups [91]. Intriguingly, *APOE* ε4 carriers had significantly higher string vessel counts than non-*APOE* ε4 carriers. However, whether these changes are primary or secondary is unknown. In addition, the OO-AD brains revealed a severe depletion of vasoactive cholinergic and noradrenergic fibers when compared to non-demented controls, potentially resulting in loss of cerebral blood flow control [91]. Incidentally, selective depletion of cholinergic cells of the nucleus basalis magnocellularis in rabbits induces cortical cholinergic deafferentation that results in Aβ deposition in the microvessels of the cerebral cortex [92]. Taken together these findings strongly suggest the participation of a dysfunctional brain microcirculation in the pathogenesis and pathophysiological evolution of AD.

In relation to the APP family of proteins, the mean amounts of APP, APP-CT99 and APLP1 were not statistically different among the 3 age groups under investigation in both the Pc and PCG with the exception of APP in the PCG in which the YA had significantly more of this protein than the MA and OO. The levels of APP-CT83 were inversely correlated with age; as age increased, the levels of this peptide decreased, suggesting that the ability to generate P3 (Aβ residues 17–42) is reduced. The APP-CTF peptides are important in the development of AD since they are usually increased and probably retained in the walls of cellular organelles and plasma membranes causing neuronal pathology and cognitive disturbances [102]–[105]. With increasing age, APLP2 in the Pc became significantly elevated while APLP2 in the PCG was significantly decreased between YA and MA. The APP family of proteins still represents a functional conundrum in the maintenance of brain homeostasis. However, these molecules are apparently involved in synapse formation and function as well as in synaptic plasticity and consolidation of memory [93], [94]. Their molecular differences and functions suggest molecular redundancy between APP, APLP1 and APLP2 supporting their importance in brain metabolism [95]. Both APLP1 and APLP2 are capable of forming dimers which appear to be important in transcellular synaptogenesis [96]. An interesting feature of the APP family is that all 3 molecules generate signal peptides upon hydrolysis of their C-terminal domains by the action of presenilin/γ–secretase. Both APP and APLP2 C-terminal fragments translocate to the nucleus while the corresponding APLP1 C-terminal peptide localizes to plasma membrane [97]. In our study, the YA cohort poses some specific challenges because these individuals died as the result of severe trauma and there was no additional information concerning their post-traumatic clinical course. Therefore, the mean holoAPP molecular levels observed in the YA Pc and PCG regions may represent a response to severe brain concussion as has been reported for acute traumatic brain injury with axonal damage [98]–[101].

Of pivotal importance in the metabolism of the APP molecule are the Aβ peptides derived by endoproteolytic action of the α-, β- and γ-secretases. In the present investigation, 29 out of a total of 78 independent ELISA estimations, comprising both regions (Pc and the PCG) in the 3 groups under study, yielded values below the detection limit of 5 pg/ml for Aβ40/Aβ42. Cases with detectable levels exhibited a spread of Aβ values with 3 cases having relatively high levels of Aβ, but still far below the low range of Aβ observed in AD. The higher amounts of Aβ in the Pc and PCG in one isolated case (#23) may be explained by the presence of mild CAA. In the remaining brain regions and age categories investigated, the Aβ levels were in accord with those observed in non-demented individuals and were compatible with the potential presence of soluble Aβ forms which are beyond microscopic detection. Among the 3 age groups that we characterized, BACE1, the secretase responsible for the N-terminal cleavage of Aβ from APP, had in quantitative terms the same pattern in both Pc and PCG with a modest elevation between YA and MA and a decrease in OO possibly ascribable to aging-related decay. This enzyme has recently been the target of intensive research which has demonstrated that its proteolytic activity is exerted on an increasing number of diverse molecules, thereby complicating the design of effective inhibitors of APP cleavage [106], [107].

Recent perspectives in understanding pathogenic factors for neurodegenerative diseases have advanced tau and α-synuclein as paradigms in which pathologically misfolded molecules lead to self- propagation and accumulation of noxious aggregates that corrupt brain homeostasis [108]. Increasing evidence suggests considerable similarity between β-amyloidosis, tauopathies and synucleopathies in neurodegenerative disorders. These molecules are capable of generating complex arrays of insoluble extracellular protein deposits and/or intracellular inclusions as well as soluble oligomeric species that eventually result in irreversible neuronal damage [109]–[115]. On the other hand, it is possible that some of these protein aggregates may have some beneficial functions [116]–[121]. Furthermore, intrinsic aging appears to induce aberrant protein conformations on molecules such as Aβ, tau and α-synuclein, which in a large number of demented individuals blend to yield complicated and/or difficult to interpret clinical outcomes. We observed a significant decrease in total tau and α-synuclein with age in the PCG, but not in the Pc. Two explanations may account for these phenomena: either there was an increasing reduction in protein synthesis (YA>MA>OO) or along aging the soluble pool of these molecules decreased because of their increasing incorporation into insoluble inclusions that escape detection in aqueous-based immunoassays.

The apolipoproteins are multifunctional proteins whose exact role in the metabolism of APP/Aβ remains to be elucidated [122]–[124]. However, in the particular context of AD, ApoE probably functions in Aβ transport [125]. In particular, the *APOE* ε4 allele represents the best known molecular risk factor for AD [126]–[128], since individuals carrying this gene in the heterozygous or homozygous conditions, will acquire sporadic AD at a younger age. However, the presence of *APOE* ε4 is not a deterministic factor for AD because many carriers will never develop this condition during their lifetimes. Intriguingly, ApoE in AD is abundantly present in both cerebrovascular and parenchymal amyloid deposits [129]. Apolipoprotein E must therefore play an important function in the vascular and parenchymal deposition of Aβ and possibly function as an *in* *situ* catalytic effector in Aβ polymerization. Moreover, ApoE may also participate in the association of Aβ with molecules of the extracellular matrix which are abundant in the amyloid deposits [130]–[133]. Our MA and OO groups were specifically selected on the basis of not being demented and having no or very low amyloid plaque scores. Hence, it was interesting to observe that the allelic frequency of *APOE* ε4 was reduced to one heterozygous individual among the 11 MA and OO participants in the study. There were no observable differences in the levels of ApoE among the 3 age groups, as determined by ELISA. Western blots of ApoJ (clusterin) demonstrated 2 bands that corresponded to ApoJ-α and ApoJ-β chains with a more abundant representation of the former. The functional dimeric ApoJ is also capable of binding and transporting Aβ peptides [125], [134]. In both the Pc and PCG, there was a noticeable increase of both isoforms in the MA group relative to the YA and a subsequent decrease in OO subjects which may be associated to age-related atrophy. On the other hand, recent studies have demonstrated that in non-demented individuals elevated CSF ApoJ correlates with a substantial degree of entorhinal cortex atrophy assessed by MRI, apparently independent from elevated p-tau, suggesting that ApoJ may hasten the progression of neurodegeneration [135].

Mild but chronic inflammation has been observed in senescent cells and the aging brain (reviewed in [33]) and neuroinflammation is common in neurodegenerative diseases. In the absence of neurodegeneration and Aβ deposition, there were no significant changes in the levels of TNF-α among the 3 groups under study. On the other hand, levels of CD200 decreased with age in the PCG. CD200 and its receptor CD200R are capable of generating anti-inflammatory signals that when deficient, as it occurs in disease or aging, may exacerbate chronic neuroinflammation. CD200 is expressed in neurons and oligodendrocytes [136], astroglia and endothelial cells [137], while CD200R is expressed in microglia [136]. CD200 and its receptor are decreased in regions with severe AD pathology [136].

In reference to the neurotrophic factors PEDF and BDNF, and the structural GFAP, our experiments revealed very little age-related fluctuation for PEDF and BDNF, although GFAP significantly increased between YA and OO age groups in the PCG. Other investigators have also shown that the presence of GFAP increases with age [33], [138]–[143]. Intriguingly, S100B demonstrated a large and statistically significant difference among the studied groups which was more evident between the YA and OO, being substantially elevated in the latter group. S100B is a multifunctional protein that, like GFAP, is relatively restricted to astrocytes and has served as a marker for brain damage in neurodegenerative diseases [144], [145]. This molecule has a deleterious role in hypoxia/ischemia and stroke where it increases gliosis, infarct expansion and proinflammatory activity [144]–[147]. Ironically, S100B also has a powerful neuroprotective function on cholinergic neurons of the nucleus basalis of Meynert during oxygen and glucose deprivation [148].

## Concluding Remarks

We quantified key molecules in the Pc and PCG brain regions which exhibit early pathological alterations in AD. In general, the majority of molecules assessed showed decreased levels in the OO group relative to YA and MA cases or remained unchanged in a few instances. Interestingly, S100B, a molecule with neuroprotective activity, exhibited substantial increases with advancing age in both PC and PCG. A salient observation was that even in two closely adjacent areas of the cerebral cortex, like the Pc and PCG, the levels of some molecules substantially differ which may be explained by anatomical and functional heterogeneity.

The kinetics and role of Aβ accumulation in the pathogenesis of AD still presents a major conundrum in understanding the clinical progress of dementia. Our results indicate that some non-demented nonagenarian individuals free of parenchymal and vascular amyloid deposits did not develop AD pathology to the same degree as that observed in demented AD subjects. Conversely, we demonstrated in a previous study that some nonagenarian individuals with a heavy load of amyloid (known as high pathology controls = HPC), similar to those commonly observed in the final stages of AD, likewise did not manifest cognitive impairment and dementia [53]. This apparent paradox implies that amyloid accumulation may be critically important in the development of cognitive failure in some individuals but might not represent the sole decisive factor for the induction of clinically manifested AD. These issues are further complicated by the recent recognition of a third group classified as suspected non-amyloid pathology (SNAP) cases in which clinical symptoms and signs of neurodegeneration are present while amyloid accumulations remain undetectable by imaging methods [149]–[151]. The cognitive failure of AD occurs when the burden of both amyloid and tangles is widespread [45]. Some observations insinuate that AD etiology is mechanistically heterogeneous and that other complicating causal conditions related to aging, in addition to Aβ, are necessary and sometimes sufficient for dementia emergence [152]. It has been recently shown that the development of AD may be linked to the nuclear loss of the repressor element 1-silencing transcription factor (REST), a molecule associated with cognitive preservation and longevity, in some neurodegenerative disorders [153]. In understanding the role of Aβ in the pathogenesis of AD, two important questions remain unanswered: 1) what are the triggering mechanisms that primarily induce Aβ deposition? and 2) why does Aβ relentlessly accumulate in such a destructive manner? The first question has not been fully addressed, although some interesting hypotheses have been advanced [116], [117], [154]–[157]. In relation to the second, recent data suggest that Aβ deposits self-propagate through the continuous accretion of misfolded and degradation-resistant molecules [108], [158]. These toxic Aβ molecules may result from structurally altered conformations, possibly induced or stabilized by posttranslational modifications common in the AD brain [159]. Furthermore, once nucleated, noxious Aβ molecules may propagate in a time-dependent fashion through cell-to-cell transmission as proposed for tau and α-synuclein (reviewed in [160]).

An interesting inference derived from our observations is that the deposition of amyloid in the OO (≥90 years of age) does not appear to be critical for the development of dementia, since these individuals can possess high plaque scores and be non-demented [53]. Dementia is clearly more associated with age-related vascular dysfunction and the spread of NFT throughout the neocortex [85]. However, there may be other factors at play, including the age-related inability to restore molecular damage, or an accumulation of a variety of other ‘lesions’, that result from the loss of, or increases in, a large number of adaptive brain processes present in most OO individuals surviving beyond the average life expectancy. Most individuals with dementia in our previous study were Braak stage V and VI [53], while those without dementia (present study) were classified as Braak stage III and IV. In addition, non-demented OO individuals exhibited a better brain perfusion preservation with lesser degrees of CAA and WMR compared to demented OO subjects [53]. To complicate matters further, neuropathological examination of individuals diagnosed with dementia of the Alzheimer’s type revealed the presence of other concurrent neuropathological lesions that by themselves are sufficient to induce the symptoms of dementia, thus confounding clinical diagnoses as well as complicating the interpretation of clinical trial outcomes [152], [161], [162].

The latest AD amyloidosis model is based on the assumption that Aβ deposition follows a time-dependent sigmoidal kinetics with an asymptotic approach to saturation virtually coincident with the onset of dementia [45]. The slope of the sigmoidal curve is likely to be variable since the deposition of amyloid in the cerebral cortex occurs at different rates and it is important to note that the early phases of the amyloid deposition processes are not well defined. It is clear that familial AD is characterized by Aβ accumulation that starts at an early age, about the third and fourth decades of life, while in sporadic AD cases the deposition of amyloid begins about the fifth and sixth decades of life. However, in neither situation is it clear that amyloid deposition progresses in a smooth, time-dependent pattern. In addition, total amyloid levels exhibit substantial variability in AD subjects and dementia itself is not strongly correlated with amyloid burden [163]. The occurrence of OO-HPC subjects reveals that amyloid deposition itself is not completely incompatible with cognitive function and suggests that a transition to dementia may be delayed or avoided. Our study adds to a body of work revealing molecular and structural heterogeneity in the development of aging and dementia and underscores the need to examine through direct observation how the processes of amyloid deposition unfold or change prior to dementia emergence.
